# Blockchain, consent and prosent for medical research

**DOI:** 10.1136/medethics-2019-105963

**Published:** 2020-05-04

**Authors:** Sebastian Porsdam Mann, Julian Savulescu, Philippe Ravaud, Mehdi Benchoufi

**Affiliations:** 1 Department of Communication, University of Copenhagen Faculty of Humanities, København, Denmark; 2 Oxford Uehiro Centre for Practical Ethics, University of Oxford, Oxford, UK; 3 Faculty of Philosophy, Oxford Uehiro Centre for Practical Ethics, Oxford, UK; 4 Mailman School of Public Health, Columbia University, New York, New York, USA; 5 Clinical Epidemiology, Université Paris Descartes Faculté de Médecine, Paris, Île-de-France, France; 6 Clinical Epidemiology, Universite Paris Descartes Faculté de Médecine, Paris, Île-de-France, France

**Keywords:** autonomy, confidentiality/privacy, informed consent, information technology, public health ethics

## Abstract

Recent advances in medical and information technologies, the availability of new types of medical data, the requirement of increasing numbers of study participants, as well as difficulties in recruitment and retention, all present serious problems for traditional models of specific and informed consent to medical research. However, these advances also enable novel ways to securely share and analyse data. This paper introduces one of these advances—blockchain technologies—and argues that they can be used to share medical data in a secure and auditable fashion. In addition, some aspects of consent and data collection, as well as data access management and analysis, can be automated using blockchain-based smart contracts. This paper demonstrates how blockchain technologies can be used to further all three of the bioethical principles underlying consent requirements: the autonomy of patients, by giving them much greater control over their data; beneficence, by greatly facilitating medical research efficiency and by reducing biases and opportunities for errors; and justice, by enabling patients with rare or under-researched conditions to pseudonymously aggregate their data for analysis. Finally, we coin and describe the novel concept of prosent, by which we mean the blockchain-enabled ability of all stakeholders in the research process to pseudonymously and proactively consent to data release or exchange under specific conditions, such as trial completion.

## Introduction

The digitalisation of medicine has led to a large increase in the types and volume of health data that could be used for research, as well as the types of analysis that can be conducted.^1^ Advances in information and communications technology have expanded the range of tools available for the secure storage, sharing and analysis of data. These trends have important implications for the traditional model of informed consent requirements, which dates back at least half a century.^2^

This contribution argues that recent work on blockchain technologies^3^ demonstrates many potential benefits of the technology across healthcare settings generally,^4–6^ and particularly in the context of consent.^7 8^ A set of advances in cryptography and mathematics which allows for a high degree of transparency and integrity in data access management, ‘blockchain technologies could be applied in the health industry in a scalable manner with high-impact results, such as improved welfare for the patients and reduced running costs for healthcare systems.’^9^ When introduced to one such blockchain-enabled infrastructure, the Massachussetts Institute of Technology’s (MIT) Open Algorithms (OPAL) framework, ‘the head of big data initiatives at the United Nations said: “This will change everything.”… The [Chief Technology Officer] of the United States Health and Human Services Department said: “Holy ***! The implications for healthcare are enormous”.’^10^

We further argue that the introduction of blockchain technologies to the healthcare context is ethically significant, because they affect one or more of the foundational bioethical principles—justice, beneficence and autonomy. In many cases, the effects will be obvious and univalent. For example, using a blockchain-based supply chain management program might reduce the circulation of counterfeit and low-quality instruments and devices through improved tracking and auditing capabilities.^11^ The effects of such a program would be to increase beneficence and justice.

However, and very importantly, the normative impacts of blockchain depend in part on the way the technology is implemented. As we argue below, a biomedical research infrastructure using blockchain for data access management and distributed computing for analysis of data stored in electronic health records has the potential to reduce the risk of privacy breaches to minimal.^10^3 Ethics and the law of most nations allow for the requirement of obtaining informed consent to be waived in cases of minimally risky research.^12^ A case could therefore be made that such an implementation of blockchain technologies would reduce the risk of all records-based research to minimal, and therefore that the requirement of informed consent should be waived for all such research. To the extent that this gets rid of selection bias and speeds up research, it has a significant positive effect on beneficence.^12^ However, by removing the option of refusing consent, this implementation would also have significant negative effects on autonomy.

The opposite case, however, could also be made. Using the cryptographic element of blockchain technologies, patients could be given complete control over who may access their medical data. They could be given the power over this access using permissions easily stored on and verifiable by a blockchain. Such an implementation would have a positive effect on patient autonomy but is likely to introduce significant selection bias, and so would likely have a strongly negative effect on beneficence.

The choice between these two implementations is not a scientific but an ethical one. Several other possible implementations of blockchain technologies likewise involve trade-offs between the bioethical principles. In the latter part of this paper, we argue that the pseudonymity and other features of blockchain networks enable new models of cooperation between stakeholders in the biomedical research ecosystem. We coin the term of *prosent* to describe the possibility that using the blockchain, patients or healthy citizens can participate in the scientific process, either by donating or selling their data to relevant research projects, by buying such data or by participating to various extent in the conduct of the research. Data exchanges based on the prosent model will likewise have very different impacts on beneficence, justice and autonomy depending on implementation. For example, should data owners be allowed or even encouraged to sell their data for profit? Which kinds of entities should be allowed to buy which kinds of data? These and many other questions are fundamentally ethical.

Historically, consent requirements have been based on bioethical principles of autonomy, justice and beneficence^2 13^ Below, we introduce blockchain technologies and argue that their implementation can be used to enhance consent procedures in ways that advance all three of these ethical goals.

## Blockchain technologies

Blockchain is a distributed technology enabling interactions of systems which, by design, does not rely on third parties to guarantee the integrity of a transaction. Instead, several features of blockchain technologies act in concert to guarantee data integrity. These are distribution of the blockchain to each member in its network, combined with a consensus mechanism designed to disincentivise fraud, and a hashing mechanism used to prove data integrity. More precisely and for convenience, we can imagine blockchain as a single shared database of which all users get a public copy, called the ledger. Table 1 lists some key principles and corresponding features and affordances of blockchain.

**Table 1:** Key features and affordances of blockchain technology

Key principles	Corresponding features	Affordances
Proof	Immutable record of transactions	Tamper-proof evidence of consent, data entry or other processes having occurred; useful for journal submissions, fraud prevention and liability concerns; supply chain management (pre/postmarket surveillance).
	Sequential timestamping	Allows proof that events happened at specific times and in specific order: for instance, tracking protocol versioning and coherence with (re)consent requirements or outcome analysis.
Differential publicity	Transparency of transactions and records	Deviations from protocol, consent, endpoints, statistical plan, and so on auditable; control over level of data visibility.
	Pseudonymity via public cryptographic identifiers	Degree of privacy can be set according to need or preference; pseudonymous identification and contact possible: prosent.
Distribution	Decentralised data access management	Accessibility of the data: control of data requests, ownership and access by patients and stakeholders are managed on the blockchain. Access of the data: data are stored off chain. Security and integrity through no database single point of failure.
	Blockchain data structure	Compatibility with distributed computing: data analytics, machine learning (federated learning, distributed secure computing, and so on).
	Consensus mechanism	Depending on the choice of the blockchain, all users or relevant stakeholders can participate in the governance and development of the blockchain, essentially on two aspects: consensus mechanism (validation nodes, proof modalities), consensus about the source code of the technology and its update.
Automation	Smart contracts	Automation of key processes (eg, claims, study recruitment, some types of data analysis, and many others), reduction of errors and fraud, integration with connected devices.

Technically, blockchains are organised in a decentralised fashion and the ledger is stored partially or in full on each of the computers (nodes) that participate in the recording and sharing of the data. Blockchains are distributed to each node in their network, are frequently updated, may be transparent and typically have low bandwidth. For these reasons, it is important to note that in most practical implementations of blockchain technologies in the healthcare context, the actual medical data of interest would not be recorded on the blockchain. Rather, the blockchain would store transactional and metadata such as hashes indicating whether or not a patient had consented; cryptographic keys denoting which healthcare professionals have access to which records; and evidence of database transactions, such as whether and when a healthcare professional has accessed a specific record, and what, if anything, that professional did with the resulting data.

For a new block to be accepted into the chain, a majority of these nodes need to agree on its veracity. This consensus mechanism is backed up by economic mechanisms designed to prevent malicious activity by disincentivising fraud.

A malignant attacker trying to corrupt data would require access to a majority (or a set, depending on the consensus mechanism) of the networked computers. This becomes almost impossible as the network grows.

### Timestamping and keeping track of events

Indeed, in a blockchain, records or data are periodically aggregated into ‘blocks’ which represent every transaction that has happened within that time frame. These blocks are linked (‘chained’) to each other using a cryptographic hash of the previous block and carry a timestamp.

Let us abstract from our argument for a moment. If we consider any transaction recorded in the blockchain as an event, then we can timestamp said event and order sequential events in time, so that we can ascertain that an event indeed happened and that of a group of events, each event happened in a precise sequence in time. This is done in a near-incorruptible way, enabling us to consistently trace events.

### Asserting and proving events

In the early history of Bitcoin technology, developers tweaked the data structure to store small pieces of information inside the blockchain. Known as a ‘hash’, this short string of characters is the result of putting a document through a hashing function. Any two identical documents will always produce an identical hash; change even a single character, and the outcome of the hashing function is radically affected. Because of these features, this little thing has important functional consequences. A hash can stand for a digital signature of any information: for instance, a document, however long, can be shortened into a hash, which then becomes its one and only ‘signature’. Thus, a person receiving a document can hash it and compare the hash to that of the original document (which in the current context is stored on the blockchain, so it cannot be altered). If the hashes match, this guarantees the integrity of the document relative to the state in which it was hashed.

In addition, each block in a blockchain contains a timestamp. Because the hash of each block includes the timestamp of the previous block, the blocks are chained together sequentially in time. In summary, hashes are digital summaries of data which are calculated on the content of each data block that makes up a blockchain. Importantly, blockchain can store as many of these proofs of data as necessary.

### Automating processes

One of the greatest promises of blockchain is related to the development of script language that enables programming on top of the blockchain architecture and so gives all the flexibility of automating processes. These pieces of code are called ‘smart contracts.’ A smart contract is essentially a piece of code which executes on the fulfilment of certain predefined, user-determined criteria. For example, a smart contract might be written to automatically upload trial data to a trials registry, if and only if certain conditions obtain: (1) that all patients have consented and (2) each phase of the trial protocol has been registered as successfully completed.

This is a potentially very powerful tool, though currently in its infancy.

### Public and private blockchains

Because blockchain technology relies on several component technologies, there are different variations on blockchains which reflect differences in the component technologies. A major distinction is between blockchains in which anyone can participate, therefore called public, and blockchains which require permissions to enter, therefore called private. The reasons for these are many and varied, but it is important to consider which architecture is best suitable for the context of healthcare broadly and research and consent in particular.

The first operational blockchain network, which underlies Bitcoin, is a public blockchain which anyone can join. It prevents fraud by forcing each computer in the node to solve hard calculations, which are calibrated to ensure a high energy cost resulting from the computational complexity. By making each computation costly in terms of resource costs, the proof of work system guarantees that messing with the previously calculated blocks becomes prohibitively expensive.

However, this dynamic is not scalable in its current form, as it consumes unacceptably large amounts of electrical energy. There are, however, other consensus mechanisms than the proof of work which are being explored but are beyond the scope of this paper.

### Data enclaves and homomorphic encryption

Distributed computing architecture may lead to the greater decentralisation of study conduct. The importance of real-world evidence and patients’ reported outcomes are in line with the contemporary sense of a need for greater patient centricity of research studies.

Blockchain architecture could help define and concretise such an infrastructure. Moreover, to open to a distributed data-sharing ecosystem with patient-level fine-grained ownership control, some research teams are designing new ways to process data. The idea is to push the algorithms rather than pull the data: algorithms process the data remotely without breaking privacy. This is especially interesting in an artificial intelligence era, where federated learning techniques would let algorithms jump from one data warehouse to another, increasing each time the spectrum of its machine-learnt knowledge.

Blockchain technologies may play an important role in these future architectures of distributed computing and federated learning. Other systems for data aggregation could be envisioned, such as safe houses or physically secure databases, also relying on public and private key cryptography. However, blockchain technologies offer several advantages, including speed of information transfer, no single target for breaches and various automations. These strong privacy-respecting systems may provoke more consent and participation to studies. For example, some entities such as the US Food and Drug Administration (FDA), and private companies, are working on a data brokerage system where a blockchain-based system would help consent to share the data to some dedicated research entities and to trace back the value created resulting from the data processing, for the purposes of distributing the value created from the analysis of data to those who have made those data available. In general, blockchain technologies could be used to manage data access, ensure transparency, reduce or prevent fraud and tampering, increase efficiency and connect stakeholders in a learning healthcare system.

### Practical consequences for the consent process

Let us sum up all these functions. Using blockchain, we can trace if and when consent was given, we can bind a consent to a document, for instance, a study protocol, on any of its versions, the proof of which are stored in the blockchain through the so-called hashes. Automaticity through smart contracts enables endless possibilities, some of which may be: automation of aspects of consent collection processes (eg, identification of potential subjects and contact via email), reconsenting being triggered when some conditions are met, for example, major changes to the protocol, and conditioning consent to feedback of results.

## Consent: autonomy

The use of blockchain technologies could give patients control over who may access their data. This would represent an increase in patient autonomy, as the patient would now be empowered to view who has permissions to access that individual’s data. The patient would also be empowered to update these permissions at will through a blockchain transaction. In effect, by interacting with a blockchain on which representations of authorisation to access data are stored, patients can easily and effectively revoke consents or grant permissions for data access. Revoking consent is technically easy and would not require special efforts from patients. At its extreme, this situation would put the individual patient in total control over their own data, since they would have the option of removing access authorisations for all or any healthcare provider.

Several such implementations exist already. We briefly introduce two of these and refer to the original papers for details.

In the Enigma model,^10^ blockchain technologies are used to manage access to data which is itself stored in a location not on the blockchain (eg, with data originators). When data are collected, it is encrypted using an encryption key shared between the data owner (ie, consenting subject) and the data acquirer (eg, the trial lead investigator). Only a hash of the original data is kept on-chain. These data can then be queried by the subject and investigator, whose identity is verified by encryption keys, using blockchain transactions.

In the Nebula model,^14^ a person wishing to access data sends a request, via a blockchain, to all relevant nodes in the network. Data are only shared if the requesting party authenticates themselves and/or permission is given by the data owner. Once authenticated, the subset of data relevant to the researcher’s study is sent automatically via smart contract to a data enclave, at which point it is deidentified to a high level of abstraction compatible with research aims and aggregated. The resulting information is then released to the researcher. Throughout this process, the data are not seen by anyone except the receiving entity, whose access to and use of data are recorded to prevent abuse.

Table 2 illustrates the potential uses of blockchain in the context of consent.

**Table 2:** Key features of blockchain technologies for implementing consent

Blockchain features	Consent
Immutable record of transactions	Record of consent cannot be subsequently altered; prevention of postfacto consent falsification; immutable record of who has accessed which information at which times; consent audit trail.

### Three uses of this system

At least three novel approaches to data sharing become possible using such a system. The first grants data subjects or originators the sole power to determine who may access their data. The second would include some default permissions on an opt-out model. The third would remove the opt-out option, thus mandating data access.

### Data owner access control

The first possibility involves giving data owners complete control over access to their data. Data owners can grant, modify or revoke permissions to access data by means of blockchain transactions. Importantly, data owners could treat different categories of data differently and assign varying levels of access protections to them. For example, access to sensitive medical data might be kept private or granted only to select entities, whereas less sensitive data might be put up for donation, or, from the point of view of some start-ups, possibly even for sale. The blockchain thus provides a practical means of implementing meta-consent.^12^

### Consent, minimal risk and default permissions

Alternatively, default settings could be set to allow certain entities access to some data. In an opt-out model, default settings would be controllable and modifiable by the data subject. It would also be possible to have certain types of data shared by default. Table 3 lists some of the potential benefits of implementation.

**Table 3:** Key features of blockchain technologies for facilitating medical research

Transparency of transactions and records	Patients, review boards, funders and other stakeholders have full overview of consent and trial status; easily auditable; accountability through visibility; may contribute to trust and efficiency; errors can be seen by all.
Pseudonomity via public cryptographic identifiers	High degree of control on privacy; level of privacy can be modulated; concerns about trial or personal conduct can be registered with high degree of privacy.
Sequential timestamping	Proof that consent was obtained before trial inclusion; proof of adherence to protocol; potential for greater patient engagement and consent due to higher trial integrity.
Decentralised storage of data	Adapt consent and prosent to decentralised nature of data generation.
Smart contracts	Conditioning of trial progression on consent; automated release of data (prevention of publication bias and knowledge silos), automate aspects of data analysis; reconsent triggered when protocol is changed; automatic warning if abnormally high levels of severe side effects are found; automatic financial or other remuneration of data subjects.
Smart contracts for secondary research	Data analysis in statistical plan can be carried out automatically; benefit sharing can be automated.

The motivation for opt-out or mandatory models stems from the effects of consent requirements on research. Consent requirements can be excessively complex, especially where the data involved are not sensitive and might be put to general, open use. They can also lead to selection bias—the systematic distortion of research results due to statistically irremediable deviations from a normal sample—which can seriously reduce the reliability of research.^15–17^

Significantly, both ethics and the law allow for consent waivers to avoid these problems if the research in question can be shown to involve only minimal risks.^18^ Using blockchain-based data access management system and multiparty secure computing could reduce the risks of much non-interventional research to minimal, since data would remain at its origin and not be subject to additional breach risks.

### Prosent: enabling bidirectional research requests

Consent protects the autonomy of patients and research subjects by allowing them to refuse unwanted treatment or participation in research. However, consent does not enable individuals to go beyond what is offered in terms of participation or interventions. Patients and research subjects might wish to exercise their autonomy by sharing other data or sharing data with other trusted research or healthcare entities. This would be possible through the prosent feature enabled by blockchain.

As manifested by the powerful trend of crowd and citizen science, many people outside the traditional research ecosystem have both the means and the willingness to gather and contribute important data.^19 20^ Indeed, citizens generally hold positive views about data sharing for public benefit research.^21 22^ Prosent could help further this trend by allowing data owners to identify each other and request data and/or participation of other citizens or scientists.

By analogy with the word consent, we propose a novel term that captures this ability: prosent. The prefix ‘con-’, originally derived from Latin cum (‘with’), refers to a joining or togetherness in the present tense. By contrast, the prefix ‘pro-’ expresses both a positive affirmation (eg, prochoice) and a forward-looking aspect (eg, prospect). Just as consent implies a current acceptance (con) of some feeling or thinking (sentio), so prosent implies a forward affirmation (pro) of an emotion or cognition (sentio).

Prosent leverages several of the affordances of blockchain to enable much greater communication between stakeholders. In a hypothetical health research ecosystem, there might be several groups of distinct stakeholders. These might include data subjects and data owners (whether individual, institutional or commercial); various data generators (including individual patients, patient advocacy groups, grassroots databases and individuals or institutions with skills in data aggregation and/or scraping); and various data acquirers (individual healthcare professionals, third-party information services, charities, both public and private institutions, hospitals and research centres; the pharmaceutical and actuarial industry; various government actors; and interested private individuals, not to mention interested loved ones).

These many stakeholders have varying degrees of motivation, insight and expertise. In the current system, few of these are leveraged; typically, the physician or institution owns the data and does not share it, except with close colleagues. However, using blockchain, it becomes possible to open up this data exchange to others who may have relevant expertise. Each entity would be represented by a pseudonymous identifier. This identifier could include information which verifies their status (eg, of healthcare professional, institution or individual) in a privacy-preserving manner, such that, though it cannot be said whom a particular identifier represents, it *can* be determined what their status is. Thus, a patient suffering from a rare disease might include information on her profile to that effect. This would enable others, whether others suffering from the same disease, or researchers interesting in advancing research, to locate a potential patient without compromising the identity of any involved.

This architecture would enable all kinds of interesting interactions between stakeholder groups. A patient with a rare disease but some resources might take it on themselves to request the data from all other patients with that rare disease who can be located pseudonymously through the blockchain. They might then issue a prosent request for the data, and, if obtained in sufficient quantities, they could then release a prosent request to an institution or individual or groups of researchers to carry out analyses on these data.

Also, fascinatingly, individuals could indicate that they wish to acquire, generate or sell data only under certain circumstances. For example, a patient suffering from an orphan disease might add an identifier to their profile, such that they can be located and contacted (still pseudonymously). Another patient might add a different kind of identifier to their profile, perhaps indicating a willingness to share their data, but only with certain entities and not others. We might imagine that many would be happy to share their data for important epidemiological work, but less enthused about doing the same (without remuneration) for commercial research. Alternatively, an individual could indicate that they only wish to share with researchers from their own social, ethnic or cultural background. Finally, monetary or healthcare incentives could be offered for making data available, although this would lead to interesting questions concerning the correct levels of regulation for a healthcare data market, and on what money can and should not buy.

Thus, we use the term ‘prosent’ to refer to the bidirectional research requests enabled by blockchain technologies. Below, we briefly sketch some of the features enabled by prosent mechanisms. This is not an exclusive list.

For one, many stakeholders who can benefit from data access but who have previously left out of the research ecosystem can exercise a greater degree of control over data; both their own data, but also by pooling or acquiring the data of others, for example, to establish a database of rare diseases. Second, these stakeholders can interact with each other pseudonymously, such that they can locate each other as entity types but not as uniquely identifiable entities and can communicate without privacy concerns. Third, various stakeholders respond to various incentives, and using prosent it is possible to offer this variety; data release could be conditioned on the aim of the study involved, the researchers or patients involved, whether or not money or healthcare has been offered and who the likely main recipients of the benefits are.

Although this is a rough description and there have been several other calls for data marketplaces, we believe the concept of prosent has not been adequately captured in the extant scholarship. The possibilities of opening up science to interested, powerful and numerous stakeholders under controllable conditions are vast. Thus, prosent, especially when coupled with smart contracts, has implications both for justice and for beneficence.

### Justice

Certain populations are under-represented in research,^23^ due to worries ranging from additional susceptibility to health risks to the ability to give voluntary consent.^24^ Others may be reluctant to trust in biomedical researchers due to historical factors.^25 26^ Still others have very rare medical conditions.^27 28^ Finally, many individuals belong to groups that are less able than others to pay for the advancement of their interests, making them less attractive targets for pharmaceutical and other medical companies.^29 30^

Prosent mechanisms offer a novel and potentially powerful means of re-engaging individuals from these communities. Using the pseudonymity of a blockchain-based, prosent-enabled data exchange, groups of individuals with similar conditions could find each other via pseudonymous profiles which could contain tags indicating the preferences and interests of that user.

### Smart contracts and beneficence

As mentioned above, smart contracts are pieces of code layered on top of a blockchain which execute automatically when certain conditions are met.

Publication bias, resulting from the preferential publication of positive results, is a known problem in biomedicine.^31^ Using smart contracts, it would be possible for stakeholders to agree at the beginning to release the data to a public trials registry, which would then happen automatically on study completion. Similarly, publicly minded patients might condition their consent on such data release, either to the public or to themselves; if such a condition were not released, the smart contract would invalidate that person’s consent.

Smart contracts could also automatically trigger a request for reconsent in cases of major protocol changes. For example, it is crucial that primary and secondary outcomes are specified in the protocol before the conduct of the study and that they are not subsequently changed or manipulated.^32^ In addition, the recording of consent on a blockchain could be fully transparent, visible and auditable for relevant stakeholders through dedicated public websites. Finally, blockchain could be used to put consent management in the hands of the patients. Patients who wish to revoke or modify their consent could do so directly via a transaction on the blockchain, without relying on a third party, such as the study administrator, to document the modification.

This is not a small opportunity, since failure to obtain or document consent is a known problem in clinical research. One review of FDA records found a failure to protect subjects and/or obtain informed consent in 53% of cases studied.^31^ Blockchain could be used to make the documentation of consent both transparent and traceable.^8^ Smart contracts could be used to freeze patient data or the progression to the next protocol phase, thus predicating the release of data on unequivocal documentation of consent.^7^

Similarly, blockchain technologies can be used to document other key components of the protocol. Any revisions to the protocol would be timestamped and transparent, reducing the incentive for fraudulent changes. These might include the data-sharing plan, the version of the analytical code used at the outset and subsequent modifications to it, and documentation of obtained consent.^7^

Looking towards the future, smart contracts could be used to automate several important bottlenecks in medical research. In theory, smart contracts in combination with secure multiparty computing and the OPAL principles could lead to a situation in which trial results are uploaded immediately on trial completion by one smart contract, and then processed and integrated to existing systematic reviews, all fully automated and much quicker than the current laborious process. Again, these suggestions are not exhaustive; smart contracts could be used in the recall and monitoring of defective drugs and medical devices; in the provenance of surgical tools; in the automated transfer of patient information to relevant healthcare providers in cases of emergency or relocation; and many more.

## Decentralisation and storage of data

In the MIT/OPAL and Enigma frameworks as well as many other worthwhile projects, a revolutionary way of preserving privacy for epidemiological research is developing. According to this paradigm, data are decentralised in the sense that it never leaves its original location. Rather, an algorithm is ‘pushed’ to the data, performs calculations on an encrypted version of those data. The aggregate calculations are then summed up to achieve an aggregate answer to a query, in which no individually identifiable information has been used at any stage. If this stage were to be combined with automatic statistical integration into the known body of medical knowledge, no human may at any time see the sensitive data, radically reducing any privacy concerns.

This contrasts to a situation in which a researcher ‘pulls’ data, that is, takes data in its raw form from many separate data sources and aggregates a database. This traditional way of doing things has some advantages, but is vulnerable to catastrophic breach risk, since any breach will affect a very large number of records.

## Future challenges

We have argued that the use of blockchain technologies can improve autonomy, justice and beneficence in biomedical research. These improvements in the biomedical research process are likely to lead to increased trust, and through trust, we may hope, greater patient engagement in research, benefiting everyone.^6^

However, several challenges need to be met before this potential can be realised.

The most salient issue is that of implementation. Blockchain technologies are novel and systems for implementing some of the above recommendations remain at the proof of concept stage.^7 8^ At the time of writing, interaction with blockchains still requires some level of cryptographic literacy. This presents a barrier to its adoption by patients and healthcare professionals. So far, there is no user-friendly solution to this problem, at least when enforcing the use of public blockchains, which we consider ought to be the default solution. The use of private blockchains should be restricted to cases of necessity.

Table 4 lists some challenges and possible solutions.

**Table 4:** Key challenges of blockchain implementation in the biomedical sciences

Challenges	Possible solutions
User-unfriendliness for identity management and user-triggered notarisation process	Many possible technical solutions; testing, trial and error; collaboration
Technical implementation: choice of architectures (public vs private blockchain or hybrid choice), complex flow to be handled by smart contracts	
Resistance to data sharing	Increasing autonomy through blockchain; moral arguments; policy and law
Legal aspects: Compliance with the EU General Data Protection Regulation (GDPR), hash personal data are pseudonymised data hence still personal, right to forget, Europe location for validator nodes	Mixing private and public blockchains; selective activation/silencing of nodes according to geographical or jurisdictional zone

In addition, the use of smart contracts will require interdisciplinary skill sets. For smart contracts to function properly, both developmental expertise and legal know-how are required. Similarly, to ensure smooth function of blockchain-based solutions, some medical professionals may have to acquire basic knowledge of the technology. To reap the full benefits of blockchain-enabled solutions, attention needs to be paid to the importance of developing such interdisciplinarity.

For ethical and methodological reason, as well as for scaling up the usage of blockchains, it is absolutely crucial that the principles behind the open source movement be embraced in this context. There are still lots of knowledge to be gained on how, for instance, to make smart contracts work properly. If the community at large agrees to cooperate and share its code and knowledge openly, progress is likely to happen in the right conditions of transparency and methodological quality and also more rapidly than if the task fell to private groups of individuals. Thus, advocacy for open source and knowledge sharing is needed for blockchain technologies to be implementable in the near future.

The implementation of blockchain technologies promises many benefits for biomedical research in general and consent procedures in particular. The whole effort now will be to move from these early ideas to actual implementation. Because this is a whole new field, these efforts will have to include significant investment in the development of necessary skills for relevant stakeholders. However, we are convinced that the fruits of such investment will be more than worth the effort.
